# *PPP1R13L*在肺腺癌脑转移瘤中的表达特征及预后研究

**DOI:** 10.3779/j.issn.1009-3419.2025.106.32

**Published:** 2025-11-20

**Authors:** LIU Luyao, FAN Peiwen, CHANG Cheng, WANG Ruozheng

**Affiliations:** ^1^830011 乌鲁木齐，新疆医科大学附属肿瘤医院新疆肿瘤学重点实验室（刘璐瑶，范佩文，王若峥）; ^1^Key Laboratory of Xinjiang Oncology, Third Clinical Medical College, Xinjiang Medical University, Urumqi 830011, China; ^2^830011 乌鲁木齐，新疆医科大学新疆地区高发疾病研究教育部重点实验室 （范佩文，王若峥）; ^2^Key Laboratory of High Incidence Diseases in Xinjiang (Ministry of Education), Xinjiang Medical University, Urumqi 830011, China; ^3^830011 乌鲁木齐，新疆医科大学附属肿瘤医院核医学科（常诚）; ^3^Department of Nuclear Medicine, Affiliated Tumor Hospital of Xinjiang Medical University, Urumqi 830011, China; ^4^830011 乌鲁木齐，新疆放射治疗临床重点专科（王若峥）; ^4^Xinjiang Clinical Key Specialty in Radiation Therapy , Urumqi 830011, China; ^5^830011 乌鲁木齐，新疆肿瘤放射治疗临床研究培育中心（王若峥）; ^5^Xinjiang Clinical Research Incubation Center for Tumor Radiotherapy, Urumqi 830011, China

**Keywords:** 肺肿瘤, 脑转移, 单细胞测序, 上皮细胞, *PPP1R13L*, Lung neoplasms, Brain metastasis, Single-cell RNA sequencing, Epithelial cells, *PPP1R13L*

## Abstract

**背景与目的:**

肺腺癌易发生脑转移，患者预后极差。蛋白磷酸酶1调节亚基13样（protein phosphatase 1 regulatory subunit 13-like, *PPP1R13L*）基因编码的p53凋亡刺激蛋白家族抑制成员（inhibitor of apoptosis-stimulating protein of p53, iASPP）蛋白是p53通路的关键抑制因子，在多种肿瘤中促癌，但其在肺腺癌脑转移中的作用未知。本研究通过单细胞测序数据整合与临床样本分析，旨在分析肺腺癌脑转移患者的肿瘤微环境特征，并探讨*PPP1R13L*在脑转移组织中的表达情况及其临床意义。

**方法:**

收集2014年1月至2024年12月新疆医科大学附属肿瘤医院就诊的4例肺腺癌脑转移和2例少突胶质细胞瘤患者的脑组织进行单细胞测序，并结合公共数据库中4例肺腺癌样本和4例正常肺组织样本的单细胞测序数据解析肿瘤微环境；另收集此医院50例肺腺癌脑转移患者的临床资料及石蜡切片，采用免疫组化技术检测iASPP蛋白表达，分析其与临床特征及预后的关系。

**结果:**

相较于胶质瘤和肺腺癌组，肺腺癌脑转移组特异的上皮细胞主要富集于氧化磷酸化、细胞凋亡、缺氧和p53通路；*PPP1R13L*作为上调差异基因，在脑转移组特异上皮细胞亚群高表达；*PPP1R13L*阳性细胞与成纤维细胞交互作用显著，激活细胞-基质黏附相关通路，关键配体-受体对为I型胶原蛋白α1链-白细胞分化抗原44（collagen type I alpha 1 chain-cluster of differentiation 44, COL1A1-CD44）。临床数据分析显示，吸烟史（HR=2.543, 95%CI: 1.159-5.583, *P*=0.020）和iASPP高表达（HR=3.351, 95%CI: 1.310-8.575, *P*=0.012）为肺腺癌脑转移患者预后的独立危险因素。

**结论:**

本研究阐明了肺腺癌脑转移微环境中上皮细胞与成纤维细胞的相互作用，提示*PPP1R13L*可作为潜在预后标志物及治疗靶点，为肺腺癌脑转移的精准治疗提供依据。

## Abstract (en)

**Background and objective:**

Lung adenocarcinoma is prone to brain metastasis, and the prognosis of patients is extremely poor. The inhibitor of apoptosis-stimulating protein of p53 (iASPP) protein, encoded by the protein phosphatase 1 regulatory subunit 13-like (*PPP1R13L*) gene, is a key inhibitor of the p53 pathway and promotes carcinogenesis in various tumors, but its role in brain metastasis of lung adenocarcinoma is unknown. This study aims to analyze the tumor microenvironment characteristics of patients with brain metastasis of lung adenocarcinoma and explore the expression of *PPP1R13L* in brain metastasis tissues and its clinical significance by single-cell sequencing and clinical sample analysis.

**Methods:**

Brain tissues from 4 patients with lung adenocarcinoma brain metastasis and 2 patients with oligodendroglioma (ODG) were collected from the Affiliated Tumor Hospital of Xinjiang Medical University from January 2014 to December 2024 for single-cell sequencing. The tumor microenvironment was analyzed by combining single-cell sequencing data from 4 lung adenocarcinoma samples and 4 normal lung tissue samples from public databases. Additionally, clinical data and paraffin sections of 50 patients with lung adenocarcinoma brain metastasis in this hospital were collected, and immunohistochemistry was used to assess iASPP expression and its association with clinicopathologic features and patient outcome.

**Results:**

Compared with the ODG and lung adenocarcinoma groups, the specific epithelial cells in the lung adenocarcinoma brain metastasis group were mainly enriched in oxidative phosphorylation, apoptosis, hypoxia, and p53 pathways. *PPP1R13L*, as an upregulated differential gene, was highly expressed in the specific epithelial cell subpopulation of the brain metastasis group; the interaction between *PPP1R13L*-positive cells and fibroblasts was significant, activating cell-matrix adhesion related pathways, with the key ligand-receptor pair being collagen type I alpha 1 chain-cluster of differentiation 44 (COL1A1-CD44). Statistical evaluation revealed that smoking (HR=2.543, 95%CI: 1.159-5.583, *P*=0.020) and high expression of iASPP (HR=3.351, 95%CI: 1.310-8.575, *P*=0.012) were independent predictors of poor prognosis in lung adenocarcinoma patients with brain metastases.

**Conclusion:**

This study revealed the interaction between epithelial cells and fibroblasts in the microenvironment of lung adenocarcinoma brain metastasis and implicate *PPP1R13L* as a potential prognostic indicator and actionable target, offering rationale for precision therapy against lung adenocarcinoma brain metastases.

作为成人最常见的颅内肿瘤类型，脑转移瘤（brain metastasis, BM）发生率约为脑原发性恶性肿瘤的10倍^[1]^。随着诊断的进步和治疗的改进，肿瘤患者生存时间显著延长，脑转移的发生亦日趋常见。BM最常见的原发肿瘤为肺癌，患者预后极差，经治后中位生存期仅为7-12个月^[2]^。流行病学研究^[3]^显示，肺腺癌（lung adenocarcinoma, LUAD）占所有肺癌脑转移病例的50.7%，显著高于鳞癌（8.3%）和小细胞癌（15.8%）。

单细胞测序以单个细胞水平的分辨率捕捉肿瘤演进过程中基因组改变与表型后果之间的动态互动，为深入理解肿瘤内部多样性开辟了新思路^[4]^。蛋白磷酸酶1调节亚基13样（protein phosphatase 1 regulatory subunit 13-like, *PPP1R13L*）基因编码的p53凋亡刺激蛋白家族抑制成员（inhibitor of apoptosis-stimulating protein of p53, iASPP）属于ASPP家族，其核心作用是拮抗p53介导的细胞凋亡^[5]^。*PPP1R13L*在多种肿瘤中高表达，可驱动肿瘤进展并提示患者预后不良^[6-8]^。

鉴于LUAD本身具有高脑转移倾向，本研究通过分析LUAD脑转移瘤（LUAD of BM, LCBM）、少突胶质细胞瘤（oligodendroglioma, ODG）和LUAD的单细胞测序数据及进行免疫组织化学技术（immunohistochemistry, IHC）验证，探究*PPP1R13L*在LCBM中的表达特征及影响患者预后的独立危险因素，为LCBM的精准治疗提供理论依据。

## 1 材料与方法

### 1.1 材料及试剂

#### 1.1.1 单细胞测序数据

为了突出转移瘤与原发脑瘤的肿瘤微环境（tumor microenvironment, TME）差异，收集本院4例LCBM和2例ODG患者脑组织样本；为了寻找转移特异性改变及定义恶性细胞的单细胞拷贝数变异（copy number variation, CNV）基线，纳入了公共数据库^[9]^（https://codeocean.com/capsule/8321305/tree/v1）中4例LUAD及4例正常对照（normal control, NC）肺组织样本的单细胞测序数据。

#### 1.1.2 石蜡切片及临床资料

本研究选取2014年1月-2024年12月于新疆医科大学附属肿瘤医院术后收集的50例LCBM组织石蜡标本及其临床资料，所有患者术前均未接受放疗或化疗。本研究已通过新疆医科大学附属肿瘤医院伦理委员会审批（伦理批号：2024BC019）。

1.1.3 IHC抗体 实验所用兔抗人iASPP多克隆抗体（货号：ab34898）购自英国Abcam公司。酶标羊抗兔IgG聚合物（PV-6000）与辣根过氧化物酶（horseradish peroxidase, HRP）标记的山羊抗兔IgG均购自北京中杉金桥有限公司。

### 1.2 方法

#### 1.2.1 单细胞捕获和建库流程

包括：（1）细胞悬液制备：对单细胞悬液进行质检，要求细胞活性高于80%。将合格的细胞洗涤后重悬，并将浓度调至700-1200 cells/μL，以备上机使用。（2）凝胶微珠乳液（gel bead-in-emulsions, GEMs）生成和Barcode标记：使用Chromium Controller系统，将质检合格的单细胞悬液、反应混合液以及带有条形码序列的5’端凝胶微珠，分别加入芯片指定的3个进样通道。（3）微反应体系构建与逆转录：通过微流控“单十字”交叉通道，将单细胞、凝胶微珠与反应混合液共同包裹于油滴内，形成GEMs微反应体系。在该体系中，细胞裂解释放mRNA，释放的mRNA与凝胶微珠上的条形码分子结合，在逆转录酶作用下合成cDNA。（4）cDNA扩增：加入Recovery Agent裂解GEMs，释放cDNA。将cDNA进行扩增、纯化和富集，使用Qubit定量试剂检测cDNA浓度，确保达到建库要求^[10]^。（5）构建5’端转录组文库和V(D)J文库：主要步骤包括cDNA片段化、末端修复、连接测序接头（Read2）以及添加样本索引（Index），最后对构建完成的文库进行定量与质检。（6）上机测序：使用NovaSeq 6000（Illumina）测序平台进行PE150双端测序，5’端转录组基因表达文库测序量至少20 k read pairs/cell，V(D)J文库测序量至少5 k read pairs/cell，同时获得每个细胞的5’端基因表达数据及免疫组库数据。

#### 1.2.2 单细胞测序的数据处理与质控

（1）质控标准：仅保留基因数在200-6000之间、线粒体基因比例低于10%的细胞，去除低质量细胞及双细胞。（2）标准化与整合：使用R语言中的“Seurat”包^[11]^对数归一化后筛选高变基因。鉴于数据来自多个样本，采用“Harmony”算法^[12]^对数据进行整合。（3）降维聚类：采用“PCA”算法降维，通过统一流形逼近与投影（uniform manifold approximation and projection, UMAP）进行可视化。依据经典标记基因注释细胞类型。（4）差异分析：使用“FindAllMarkers”函数^[13]^识别各细胞亚群中的差异表达基因（|logFC|>1且校正*P*<0.05），并结合经典细胞标记基因进行手动注释。所有分析均使用*Wilcoxon*检验。

#### 1.2.3 富集分析

为系统解析差异表达基因的生物学功能及其参与的信号通路，本研究通过“clusterProfiler”^[14]^和“msigdbr”^[15]^包进行基因本体论（Gene Ontology, GO）、京都基因与基因组百科全书（Kyoto Encyclopedia of Genes and Genomes, KEGG）和Hallmark通路的富集分析。

#### 1.2.4 CNV

采用“infercnv”包^[16]^，以NC细胞为参照，推断LUAD及LCBM上皮细胞的CNV。

#### 1.2.5 拟时序分析（pseudo-time analysis, PTA）

采用“Monocle3”^[17]^进行单细胞轨迹推断，所有参数保持默认。

#### 1.2.6 细胞间通讯分析

通过R软件包“CellChat”^[18]^完成，数据库为CellChatDB.human，保留在≥5%细胞中表达的配体-受体对；使用“computeCommunProb”和“filterCommunication”进行显著性筛选，并通过内置可视化函数展示通讯网络及关键信号变化。

#### 1.2.7 生存分析

“KM plotter”^[19]^用于*Kaplan-Meier*生存分析；“survival”（https://CRAN.R-project.org/package=survival）用于绘制森林图。本研究的主要研究终点为LCBM患者的总生存期（overall survival, OS）。OS定义为从经影像学检查确诊发生脑转移之日起，至因任何原因死亡或末次随访之日止的时间间隔。本研究随访截止日期为2025年6月30日，随访时间范围为0.6-116个月，中位随访时间为33.3个月

#### 1.2.8 IHC

患者石蜡切片经脱蜡、脱苯及梯度水化处理，高温抗原修复，冷却后用IHC检测LCBM组织中iASPP的表达水平，阴性对照以磷酸盐缓冲液替代一抗。实验结果通过阳性细胞比例与染色强度两项指标进行判定。阳性细胞百分率即标本在高倍镜随机5个视野中分别计200个细胞中含有棕黄色颗粒的细胞所占比例（<5%为0分；5%-25%为1分；26%-50%为2分；51%-75%为3分；>75%为4分）；染色强度即根据阳性细胞中棕黄色颗粒的颜色深浅进行评分（无染色为0分；弱染色为1分；中染色为2分；强染色为3分）。两者乘积作为最终判定标准，对癌组织中iASPP表达进行评分，以≤4分作为iASPP低表达组，判定为iASPP阴性表达；≥6分作为iASPP高表达组，判定为iASPP阳性表达^[20]^。人类蛋白质图谱数据库（human protein atlas, HPA）（https://www.proteinatlas.org/）展示iASPP在LUAD中的表达情况。

### 1.3 统计学方法

分类变量采用卡方检验或*Fisher*精确检验分析iASPP表达与临床特征的关系；等级资料采用秩和检验进行分析。采用*Spearman*秩相关分析上皮细胞PPP1R13L与*CD44*的相关性。采用*Kaplan-Meier*法及*Log-rank*检验、单因素和多因素*Cox*回归分析方法探讨吸烟史、iASPP表达等与预后的关系。*P*<0.05为差异具有统计学意义。

## 2 结果

### 2.1 单细胞图谱揭示LCBM独特细胞组成及*PPP1R13L*潜在预后价值

通过对新疆医科大学附属肿瘤医院的4例LCBM和2例ODG患者的脑组织进行单细胞测序，并将公共数据库中4例LUAD样本的单细胞测序数据进行整合，分为LCBM、ODG和LUAD共3组。降维聚类后注释为7种主要的细胞类型：T/自然杀伤（natural killer, NK）细胞、胶质细胞、髓系细胞、内皮细胞、成纤维细胞、上皮细胞、B细胞（图1A）。细胞组成分析显示，LCBM组较ODG组显著富集上皮细胞，较LUAD组显著富集胶质细胞（图1B、1C）。Hallmark通路分析结果（图1D）显示，与ODG组和LUAD组相比，p53和核因子-κB（nuclear factor-κB, NF-κB）信号通路在LCBM组织中均呈现显著富集（*P*<0.05）。通过对比富集于p53和NF-κB信号通路的基因，筛选出其中的交集基因——*PPP1R13L*（图1E）。相较于ODG和LUAD组，该基因在LCBM组显著上调，且主要在上皮细胞高表达（图1F、1G）。因此推测，*PPP1R13L*可能通过p53/NF-κB通路促进LUAD脑转移。

**图 1:**
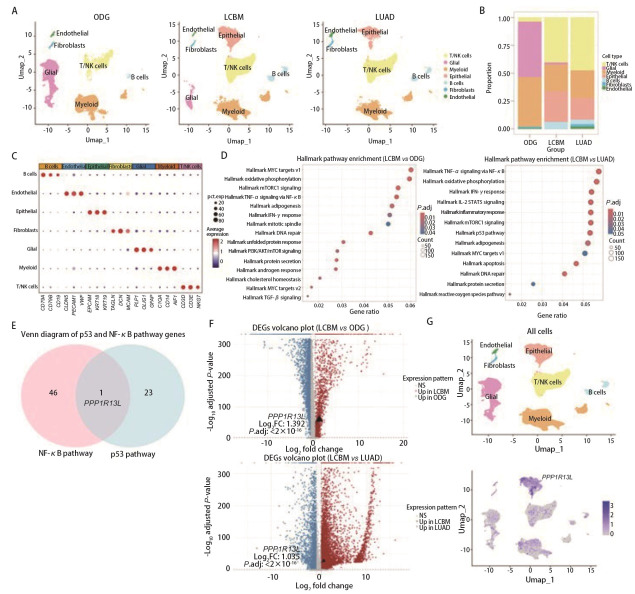
单细胞图谱揭示LCBM独特细胞组成及PPP1R13L潜在预后价值。A：ODG、LCBM和LUAD组的细胞类型分群UMAP图；B：3组中不同细胞类型占比图；C：每种细胞类型的标记基因表达，其中点大小和颜色代表标记基因的百分比；D：LCBM组对比ODG组（左）和LUAD组（右）的Hallmark通路分析气泡图；E：LCBM上调基因在NF-κB和p53信号通路中的富集分析韦恩图；F：LCBM和ODG（上）及LUAD（下）差异基因表达火山图；G：PPP1R13L在不同细胞类型上的表达UMAP图。

### 2.2 鉴定LCBM特异性高恶性上皮细胞亚群及其代谢重编程特征

由于ODG组未见上皮细胞富集，纳入了4例NC样本进行下一步分析。将所有上皮细胞降维聚类后得到7个亚群，通过对比统一流形逼近与投影（uniform manifold approximation and projection, UMAP）图（图2A）和细胞比例图（图2B）发现，第0、3簇细胞仅在LCBM组存在，而在NC和LUAD组未观察到，提示第0、3簇细胞可能促进脑转移。

**图 2:**
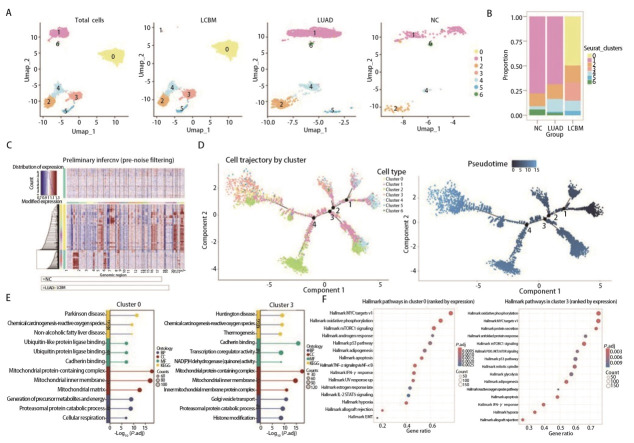
鉴定LCBM特异性高恶性上皮细胞亚群及其代谢重编程特征。A：所有细胞、LCBM、LUAD和NC的上皮细胞分簇UMAP图；B：3组中不同簇的细胞占比图；C：上皮细胞拷贝数变异分析热图。横坐标为染色体位置（按基因组坐标排列），纵坐标为不同肿瘤类型（LCBM和LUAD），颜色梯度代表相对拷贝数变化：红色表示扩增（gain），蓝色表示缺失（loss），颜色深浅与变异幅度成正比；对照参考集为NC组上皮细胞簇；D：上皮细胞的分化轨迹，每种颜色都编码簇（左）和伪时间（右）；E：第0簇（左）和第3簇（右）细胞的GO和KEGG富集分析；F：第0簇（左）和第3簇（右）细胞的Hallmark通路分析。

为了定义恶性细胞的CNV基线，接下来以NC组的上皮细胞簇为参照细胞，对LUAD组和LCBM组的上皮细胞簇进行CNV。结果（图2C）显示，与LUAD组相比，LCBM组在多个基因组区域呈现更显著的拷贝数变异，恶性程度更高。拟时序分析（图2D）进一步表明第0、3簇处于分化尾端，证实其为高恶性上皮亚群。功能富集分析（图2E）表明，第0、3簇细胞均显著富集于氧化应激、线粒体功能、蛋白酶体降解等过程。Hallmark通路分析（图2F）提示两簇均与代谢重编程（氧化磷酸化、糖酵解）、增殖相关通路（如MYC和mTORC1信号）及p53通路有关。

### 2.3 *PPP1R13L*阳性细胞的功能

为探究*PPP1R13L*在LUAD脑转移中的作用，分析了其在不同肿瘤中的表达特征，结果（图3A）显示，相较于ODG组，*PPP1R13L*在LCBM组中的表达水平显著升高（*P*<0.05）。进一步聚焦上皮细胞亚群发现，相较于LUAD组，LCBM组中*PPP1R13L*的表达量显著上调（*P*<0.05）；LCBM组*PPP1R13L*的表达量较NC组升高，但差异无统计学意义（*P*>0.05）。*PPP1R13L*在上皮细胞亚群中的表达模式图（图3B）表明其主要富集于第3簇上皮细胞。

**图 3:**
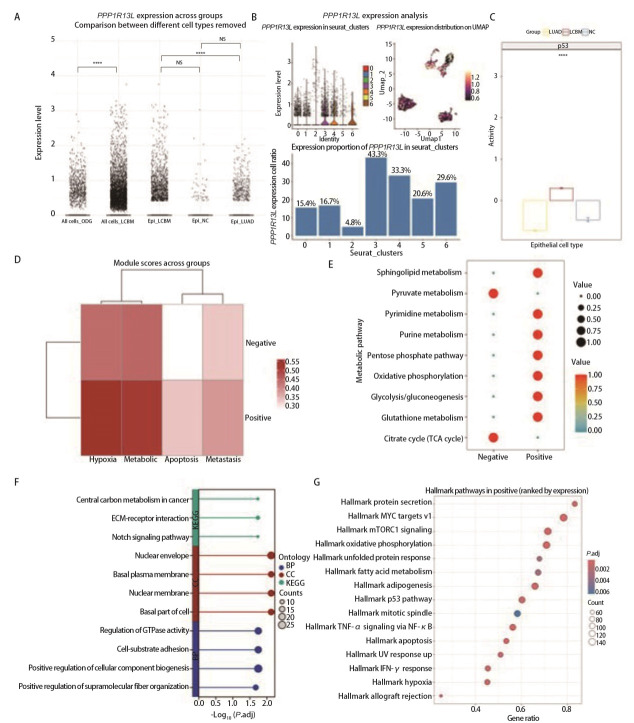
PPP1R13L阳性细胞的功能。A：PPP1R13L在NC、LUAD及LCBM间的表达差异统计图；B：PPP1R13L在上皮细胞中各簇的表达情况；C：p53信号通路在3组中的表达情况；D：PPP1R13L阳性/阴性细胞功能模块评分热图；E：PPP1R13L阳性/阴性细胞的代谢通路活性气泡图；F：PPP1R13L阳性组差异基因的GO/KEGG通路富集棒棒糖图；G：PPP1R13L阳性细胞的Hallmark通路分析。

鉴于*PPP1R13L*与p53通路的已知关联，检测了各组上皮细胞的p53通路活性，结果显示LCBM组上皮细胞的p53通路活性显著高于LUAD和NC组，且与*PPP1R13L*表达趋势一致，提示其可能通过调控p53通路促进转移（图3C）。进一步将LCBM上皮细胞按*PPP1R13L*表达量分组，发现阳性组在缺氧应答、代谢活性、凋亡及转移相关表型上更为显著，氧化磷酸化（oxidative phosphorylation, OXPHOS）和糖酵解等代谢通路明显激活（图3D、3E）。

富集分析（图3F）显示，阳性组差异基因富集于碳代谢、细胞外基质（extracellular matrix, ECM）互作、Notch信号通路及细胞-基质黏附等过程。Hallmark通路分析（图3G）进一步表明，*PPP1R13L*阳性细胞显著富集于p53、凋亡、代谢重编程相关通路及增殖相关通路，与第0、3簇上皮细胞的特征高度一致，表明*PPP1R13L*可能通过协同调控p53与代谢重编程，推动LCBM进展。

### 2.4 *PPP1R13L*通过I型胶原蛋白α1链-白细胞分化抗原44（collagen type I alpha 1 chain-cluster of differentiation 44, COL1A1-CD44）信号轴参与上皮-成纤维细胞互作

为进一步探究上皮细胞与其他细胞类型之间的相互作用及其在细胞-基质黏附中的作用开展了细胞通讯分析。结果显示，上皮细胞是互作网络的核心枢纽（图4A）；细胞间通讯显示，阳性组与成纤维细胞的互作差异最为显著（图4B）。通过配体-受体分析鉴定出关键互作对COL1A1-CD44（图4C），进一步验证（图4D）显示，相较于*PPP1R13L*阴性组，COL1A1与CD44在阳性组中均呈高表达。相关性分析结果（图4E）表明，*PPP1R13L*与CD44存在弱的正相关关系。成纤维细胞的富集分析（图4F）显示，差异基因显著富集于细胞连接、细胞-基质黏附以及ECM重塑等生物学过程。上述结果表明，*PPP1R13L*可能通过调控COL1A1的表达，激活CD44信号，进而诱导成纤维细胞的功能重塑，最终影响TME的维持与演进。

**图 4:**
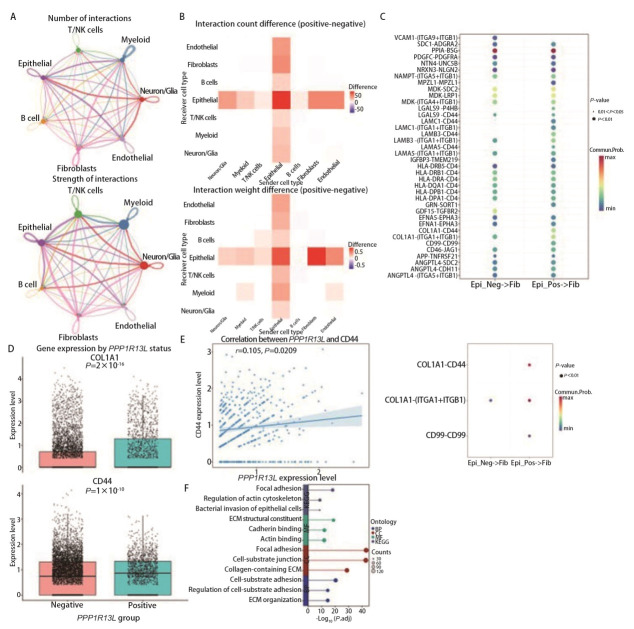
PPP1R13L通过COL1A1-CD44信号轴参与上皮-成纤维细胞互作。A：不同细胞类型相互作用数量（上）和强度（下）网络图；B：不同细胞类型相互作用数量（上）和强度（下）差异热图；C：PPP1R13L阴性/阳性组上皮与成纤维细胞之间配体-受体相互作用对通信概率的影响（放大前后）；D：PPP1R13L阴性/阳性组上皮细胞上COL1A1（上）与CD44（下）的表达量；E：上皮细胞PPP1R13L与CD44的相关性分析；F：成纤维细胞的GO和KEGG富集分析。

### 2.5 LCBM患者脑组织中iASPP表达与预后的关系

纳入新疆医科大学附属肿瘤医院50例LCBM患者，收集其临床资料（表1）并采用IHC检测脑组织中iASPP的表达（图5A），结合HPA数据库中LUAD典型病例的对照，结果显示iASPP在LCBM中的表达高于LUAD。依据评分将患者分为iASPP高、低表达组，并将其与各项临床特征进行分析，结果表明，iASPP表达水平与患者年龄、性别等临床特征的关联均不明显（*P*>0.05）（图5B）。

**表 1:** 50例LCBM患者的基线特征及预后因素分析（*n*=50）

Features	Group	*n* (%)	Univariate analysis			Multivariate analysis	
			HR (95%Cl)	*P*		HR (95%Cl)	*P*
Gender	Male	31 (62.0)	Reference				
	Female	19 (38.0)	0.594 (0.249-1.417)	0.241			
Age (yr)	≤60	27 (54.0)	Reference				
	>60	23 (46.0)	1.184 (0.548-2.558)	0.668			
Pulmonary tuberculosis	None	33 (66.0)	Reference			Reference	
	Yes	17 (34.0)	2.030 (0.924-4.463)	0.078		1.566 (0.693-3.538)	0.281
Intracranial symptoms	None	17 (34.0)	Reference				
	Yes	33 (66.0)	1.816 (0.755-4.368)	0.183			
Extracranial metastasis	None	13 (26.0)	Reference				
	Yes	37 (74.0)	1.955 (0.734-5.206)	0.180			
Smoking	None	33 (66.0)	Reference			Reference	
	Yes	17 (34.0)	2.540 (1.171-5.506)	0.018		2.543 (1.159-5.583)	0.020
KPS score	≥90	22 (44.0)	Reference				
	70-89	24 (48.0)	1.604 (0.683-3.768)	0.278			
	<70	4 (8.0)	0.814 (0.166-3.988)	0.799			
T stage	T1-T2	32 (64.0)	Reference				
	T3-T4	15 (30.0)	1.237 (0.523-2.925)	0.628			
	Unknown	3 (6.0)	3.196 (0.703-14.519)	0.133			
N stage	N0-N1	13 (26.0)	Reference				
	N2-N3	34 (68.0)	1.576 (0.584-4.251)	0.369			
	Unknown	3 (6.0)	2.823 (0.539-14.786)	0.219			
Number of BM	Single	15 (30.0)	Reference			Reference	
	Multiple	35 (70.0)	2.725 (0.933-7.956)	0.067		2.226 (0.730-6.793)	0.160
Size of BM (cm)	≤3	24 (48.0)	Reference				
	>3	26 (52.0)	0.594 (0.266-1.325)	0.203			
EGFR	Wild	22 (44.0)	Reference				
	Mutant	28 (56.0)	0.850 (0.391-1.848)	0.682			
iASPP	Low	40 (80.0)	Reference			Reference	
	High	10 (20.0)	2.873 (1.150-7.179)	0.024		3.351 (1.310-8.575)	0.012

KPS: Karnofsky performance status; EGFR: epidermal growth factor receptor; iASPP: inhibitor of apoptosis-stimulating protein of p53; HR: hazard ratio; CI: confidence interval.

**图 5:**
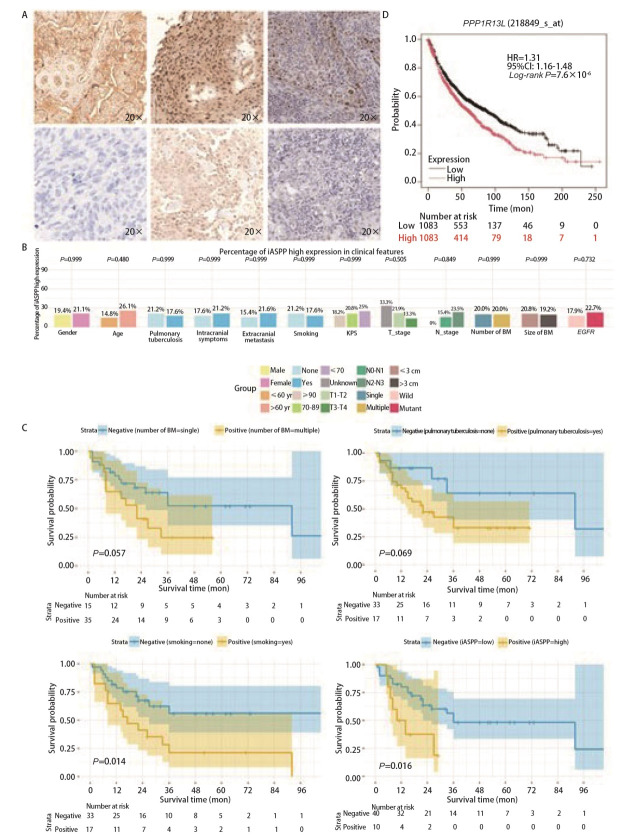
LUAD脑转移患者脑组织中iASPP表达与预后的关系。A：iASPP在不同癌组织中的表达。代表性免疫组化图像显示：阳性对照（左上）、阴性对照（左下）、LCBM高表达（上）、LCBM低表达（下）、LUAD高表达（右上）和LUAD低表达（右下）；B：临床特征与iASPP的关系；C：LUAD脑转移瘤组织中脑转移瘤数量、肺结核史、吸烟史、iASPP表达量的Kaplan-Meier生存曲线；D：PPP1R13L在TCGA-LUAD中的生存分析图。

以患者OS为观察终点，死亡为结局事件，对各项临床特征进行预后分析，iASPP低表达组中位OS为21.5个月，高表达组中位OS为10.7个月。单因素Cox回归结果（表1）显示，肺结核史、吸烟史、BM数量及iASPP表达与OS有关（*P*<0.10）；将上述变量纳入多因素Cox回归分析，进一步确定吸烟史（HR=2.543, 95%CI: 1.159-5.583, *P*=0.020）和iASPP高表达（HR=3.351, 95%CI: 1.310-8.575, *P*=0.012）为患者预后的独立危险因素。进一步进行*Kaplan-Meier*分析（图5C）显示，吸烟史和iASPP高表达均与不良预后显著关联（P<0.05）。为了验证iASPP编码基因*PPP1R13L*在LUAD中的临床意义，利用癌症基因组图谱（The Cancer Genome Atlas, TCGA）数据库中的LUAD队列进行生存分析（图5D），结果显示*PPP1R13L*高表达与LUAD患者的不良预后显著关联（*P*<0.05）。

## 3 讨论

BM在诊断时的发病率为10%-20%，在病程中的发病率约为25%^[21]^。尽管诊疗手段不断进步，但肿瘤细胞与TME间的复杂相互作用仍需深入研究，以改善预后预测和治疗效果。

iASPP通过抑制p53的转录活性，阻断了抗肿瘤免疫应答，从而促进肿瘤的免疫逃逸与增殖^[22]^。*PPP1R13L*在结直肠癌^[23]^、宫颈癌^[24]^及胃癌^[25]^中的作用已有研究，但在LCBM过程中的功能及机制尚未阐明。本研究整合单细胞转录组与临床病理数据，探讨*PPP1R13L*在LCBM进展及预后中的作用。

通过单细胞转录组分析显示，LCBM以上皮细胞和胶质细胞显著富集为主要特征，随后鉴定出*PPP1R13L*在p53和NF-κB通路中共同富集，且主要在上皮细胞高表达，体现了转移瘤与原发性肿瘤在细胞起源和TME方面的根本差异^[26]^。尽管*PPP1R13L*被认为是p53的负调控因子，本研究却观察到p53通路活性在LCBM组上调，这一现象可从缺氧环境下p53的调控机制中得到解释：在缺氧条件下，MDM2活性受到抑制，p53大量积累并且发生核转位，与缺氧诱导因子-1α（hypoxia-inducible factor 1-alpha, HIF-1α）竞争性结合转录共激活因子p300，导致p53及其下游靶基因表达上调^[27]^；中度缺氧时，HIF-1α被激活，通过诱导糖酵解和诱导PNUTS促进p53的部分积累^[28]^。已有研究在结直肠腺癌^[29]^和甲状腺乳头状癌^[30]^中观察到HIF-1α与p53表达呈正相关。这与Wang等^[24]^发现*PPP1R13L*表达与p53通路、缺氧反应及磷脂酰肌醇3-激酶/蛋白激酶B/哺乳动物雷帕霉素靶蛋白（phosphatidylinositol 3-kinase/protein kinase B/mammalian target of rapamycin, PI3K/AKT/mTOR）通路呈显著正相关一致。因此，*PPP1R13L*上调与p53通路活性升高并不矛盾，而是反映了在缺氧、DNA损伤等应激场景下，*PPP1R13L*同步上调以抑制其过度激活，最终导向促存活而非凋亡的细胞命运。

本研究的核心创新在于揭示了*PPP1R13L*在LCBM中的关键作用：*PPP1R13L*阳性上皮细胞表现出更强的代谢活性、缺氧应答、抗凋亡及转移表型，且与p53通路活性有关。本研究证实了肿瘤细胞在转移过程中通过重塑TME实现免疫逃逸的假说，并揭示了代谢重编程^[31]^、缺氧^[32]^与转移过程有关。基于已有综述^[33]^明确提示OXPHOS是乳腺癌脑转移的重要代谢适应机制，本课题组推测该代谢通路在LCBM中可能发挥相似功能，这为后续机制探索提供了方向。此外，细胞通讯结果提示*PPP1R13L*可能参与了细胞-基质黏附：*PPP1R13L*阳性细胞与成纤维细胞互作显著，COL1A1-CD44为关键配体-受体对。Lin等^[34]^指出，CD44与iASPP相互作用影响成纤维细胞迁移和存活，提示*PPP1R13L*通过多途径协同促脑转移。因此本课题组推测，*PPP1R13L*阳性肿瘤细胞高表达CD44，结合成纤维细胞表面的COL1A1，激活细胞-基质黏附和相关信号通路，导致ECM重塑，这与既往研究^[35]^高度一致。

近年来，关于LCBM的治疗已从全脑放疗转向靶向-免疫-代谢联合干预。在靶向治疗新靶点方面，Huang等^[36]^发现CD44^+^周细胞样细胞通过GPR124增强的跨内皮迁移引起脑转移，提示阻断CD44可削弱转移前微环境形成。针对CD44的单克隆抗体（如RG7356）已进入I期临床试验，证实对晚期实体瘤患者具有一定的疾病控制率^[37]^。在肿瘤转移过程中，ECM的重塑和降解促进了肿瘤细胞的侵袭和迁移，从而促进了疾病的进展^[38]^。因此，针对ECM的治疗策略已被设计为通过干预这一复杂过程来阻碍肿瘤转移。在免疫治疗策略上，与传统化疗提升患者PFS相比，程序性细胞死亡受体-1（programmed cell death protein-1, PD-1）抑制剂和细胞毒性T淋巴细胞相关蛋白-4（cytotoxic T-lymphocyte-associated protein-4, CTLA-4）治疗已成热点^[39,40]^。既往研究^[41]^显示，接受PD-1抑制剂治疗时，转移负荷与转移部位是影响免疫疗效及预后的重要因素。在代谢干预领域，Faubert等^[31]^指出肿瘤在转移过程中会动态切换代谢需求，导致原发灶与转移灶出现不同耐药表型。Liu等^[42]^证实LCBM细胞呈谷胱甘肽高消耗状态，抑制GPX4-铁死亡轴可显著增强铂类药物抗癌作用。Wang等^[43]^已证实脂质代谢异常在肺癌早筛中的价值。综上，BM特有的代谢重编程可能是一种有前景的治疗策略。

依据既定评分标准评估iASPP表达后，统计学分析结果显示，iASPP表达水平在性别、年龄、吸烟史、表皮生长因子受体（epidermal growth factor receptor, *EGFR*）突变等临床特征方面无统计学差异（*P*>0.05），与Wang等^[44]^研究结果一致。多因素*Cox*回归分析表明，iASPP高表达与吸烟史共同作为影响患者OS的独立危险因素，这一发现与当前学界认知高度吻合：一方面，吸烟史^[45]^已被证实是驱动脑转移的关键临床风险因子；另一方面，iASPP^[46]^也被报道通过促进mTOR依赖自噬促进肿瘤生长，且与不良预后有关。国内研究在胶质瘤^[47]^及宫颈癌^[48]^中同样发现iASPP与p53蛋白表达呈正相关。本研究将这两个因素关联起来，揭示了它们在LCBM中的协同作用机制，*Kaplan-Meier*生存曲线进一步证实二者与患者的不良预后有关。这一发现凸显iASPP作为LCBM潜在预后生物标志物的临床价值^[49]^，也与TCGA生存分析显示其高表达与不良预后关联的结果一致。

本研究存在一定的局限性：首先，纳入分析的单细胞测序样本数量相对较少，尽管整合了公共数据库，但较小的样本量可能无法完全捕捉LCBM组TME的高度异质性，某些“特异性”细胞亚群可能受个体差异影响，这在一定程度上带来了潜在的偏倚风险，后续将会在更大规模的队列中验证；其次，本研究为单细胞转录组层面分析，未能对p53及关键基因的突变状态进行检测，限制了我们对p53通路调控机制的深入解析；最后，本研究主要集中于发现和描述现象，相关机制结论为基于生物信息学的初步推测，后续我们将通过体内外功能实验进行进一步验证。

综上所述，本研究从单细胞层面初步揭示了LCBM的TME异质性，提示了*PPP1R13L*在细胞内通过抑制p53的凋亡和细胞外经COL1A1-CD44轴重塑基质微环境的双重作用促进脑转移进程的可能性，这些发现为针对*PPP1R13L*及其下游通路COL1A1-CD44轴的开发奠定了坚实的理论基础。
